# Perinatal exposure to UDCA prevents neonatal cholestasis in *Cyp2c70*^*-/-*^ mice with human-like bile acids

**DOI:** 10.1038/s41390-022-02303-5

**Published:** 2022-09-23

**Authors:** Hilde D. de Vries, Anna Palmiotti, Rumei Li, Milaine V. Hovingh, Niels L. Mulder, Martijn Koehorst, Vincent W. Bloks, Tim van Zutphen, Folkert Kuipers, Jan Freark de Boer

**Affiliations:** 1 Department of Laboratory Medicine, University Medical Center Groningen, University of Groningen, Groningen, The Netherlands; 2 Faculty Campus Fryslân, University of Groningen, Leeuwarden, The Netherlands; 3 Department of Pediatrics, University Medical Center Groningen, University of Groningen, Groningen, The Netherlands

## Abstract

**Background:**

*Cyp2c70*^*-/-*^ mice with a human-like bile acid (BA) composition display features of neonatal cholestasis. We assessed whether perinatal ursodeoxycholic acid (UDCA) exposure prevents neonatal cholestasis in *Cyp2c70*^*-/-*^ mice and reduces cholangiopathy development later in life.

**Methods:**

*Cyp2c70*^*+/-*^ males were crossed with *Cyp2c70*^*+/-*^ females fed either a regular chow diet or a 0.1% UDCA-containing diet during breeding, gestation, and suckling. Cholestasis and liver function parameters were assessed in their *Cyp2c70*^*-/-*^ and wild-type offspring at 3 and 8 weeks of age.

**Results:**

Three-week-old *Cyp2c70*^*-/-*^ pups showed features of neonatal cholestasis, including elevated plasma BAs and transaminases, which were completely prevented in *Cyp2c70*^*-/-*^ pups upon perinatal UDCA exposure. In addition, UDCA administration to the dams corrected altered hepatic gene expression patterns in *Cyp2c70*^*-/-*^ pups, reduced markers of fibrogenesis and inflammation, and prevented cholangiocyte proliferation. Yet, these beneficial effects of perinatal UDCA exposure were not retained into adulthood upon discontinuation of treatment.

**Conclusion:**

Perinatal exposure of *Cyp2c70*^*-/-*^ mice to UDCA has beneficial effects on liver function parameters, supporting a direct role of BA hydrophobicity in the development of neonatal cholestasis in these mice. However, prevention of neonatal cholestasis in *Cyp2c70*^*-/-*^ mice has no long-lasting effects on liver pathophysiology.

**Impact:**

- This is the first study showing that perinatal UDCA exposure prevents features of neonatal cholestasis that are observed in mice with a human-like bile acid composition, i.e., *Cyp2c70*^-/-^ mice.
- Perinatal UDCA exposure of *Cyp2c70*^*-/-*^ pups leads to UDCA enrichment in their circulating bile acid pool and, consequently, to a reduced hydrophobicity of biliary bile acids.
- Perinatal UDCA exposure of *Cyp2c70*^*-/-*^ pups has no long-lasting effects on the development of cholangiopathy after discontinuation of treatment.
- The results in this study expand current knowledge regarding acute and long-lasting effects of UDCA treatment in early life.

## Introduction

Bile acids (BAs) are amphipathic molecules that are synthesized in the liver, stored in the gallbladder, and released into the intestine upon ingestion of a meal to facilitate lipid absorption. Due to their lipid-solubilizing properties, high intra- and extracellular concentrations of BAs can cause liver damage.^1^ BAs also act as hormones through activation of several nuclear and membrane-bound receptors and thereby modulate metabolism and immune functions.^2^ Elevated plasma BA concentrations are frequently observed in human newborns, without evident underlying pathological conditions.^3^ In most cases, BA levels decrease within two weeks without further intervention. This relatively benign phenomenon, sometimes referred to as “physiological neonatal cholestasis”, is hypothesized to be caused by an underdeveloped BA transport system.^4–6^

Cholestasis is characterized by impaired bile flow, leading to the accumulation of bile constituents, including BAs, in the liver and blood.^7^ In case of persisting symptoms, neonatal cholestasis can result in severe liver disease. Various underlying causes have been described, ranging from genetic defects in BA synthesis and transport enzyme systems to viral infections and blockage or malformation of bile ducts.^8^

Ursodeoxycholic acid (UDCA), a hydrophilic BA, is often used for the treatment of various cholestatic liver diseases.^9^ UDCA is proposed to improve cholestasis by stimulating bile canalicular efflux pumps as well as by reducing the hydrophobicity and, hence, the cytotoxic potential of the BA pool.^10^ Furthermore, UDCA is hypothesized to stimulate bicarbonate secretion from cholangiocytes, which creates an alkaline environment at the apical membrane. This so-called “bicarbonate umbrella” protects cholangiocytes from cytotoxic molecules such as hydrophobic BAs.^11^ In addition, UDCA is reported to have cytoprotective, anti-inflammatory, antifibrotic,^12^ and endoplasmic reticulum (ER) stress-reducing properties.^13^ UDCA is FDA-approved for the treatment of primary biliary cholangitis in adults and is applied to alleviate pruritus in pregnant women diagnosed with intrahepatic cholestasis of pregnancy.^14^

BA metabolism, as well as the consequences of disturbance of BA homeostasis, has been extensively studied in mouse models despite the abundant presence of specific murine BAs that are absent in humans.^15^ Unlike the hydrophobic BA species that are abundantly present in humans, these murine BA species, i.e., the primary α-, β- and the secondary ω-muricholic acids (MCAs), are extremely hydrophilic and have cytoprotective rather than cytotoxic effects.^16,17^ Furthermore, MCAs have limited capacity to promote lipid absorption and exert antagonistic activity toward the BA receptor farnesoid X receptor (FXR/NR1H4),^18^ which is in sharp contrast to the FXR activating BA species that are abundant in the human pool.^19,20^ In order to improve the translational potential of pre-clinical data to humans, mouse models with a more human-like BA composition are essential. Recently, CYP2C70 was demonstrated to be responsible for the synthesis of α- and β-MCA from chenodeoxycholic acid (CDCA).^21,22^ Since then, we and others have generated *Cyp2c70*-deficient mice that lack MCAs and, consequently, have a more hydrophobic, human-like, BA profile consisting of mainly cholic acid, CDCA, deoxycholic acid, and lithocholic acid.^23–25^

*Cyp2c70*-deficient mice show features of “physiological neonatal cholestasis”, characterized by very high plasma BA and transaminase levels at weaning,^23^ that may initiate a cascade of events that contributes to the acceleration of liver pathology in Cyp2c70-KO mice. Although plasma BA and transaminase concentrations in *Cyp2c70*^*-/-*^ mice drop markedly between the age of 3 and 12 weeks, particularly adult female *Cyp2c70*^*-/-*^ mice develop cholangiopathy with bridging fibrosis in adulthood that can be prevented by UDCA treatment.^23^ We hypothesized that UDCA exposure at an early age would prevent characteristics of neonatal cholestasis in *Cyp2c70*-deficient mice and thereby delay or (partly) prevent the development of liver fibrosis in later life. Therefore, we fed female *Cyp2c70*^*+/-*^ mice a diet supplemented with 0.1% UDCA during pregnancy and suckling and evaluated parameters of cholestasis and liver pathophysiology in their *Cyp2c70*^*-/-*^ offspring during the suckling period as well as in young-adult mice 4 weeks after they had been weaned to a non-UDCA-containing diet.

## Materials and methods

### Animals and diet

*Cyp2c70*^+/-^ male and female mice on a C57BL/6J background (C57BL/6J-Cyp2c70^em3Umcg^) were used for breeding, generating wild-type (WT), *Cyp2c70*^*+/-*^ and *Cyp2c70*^*-/-*^ offspring, the latter having a human-like BA pool that is devoid of MCAs.^23^

*Cyp2c70*^+/-^ dams received either normal chow (Ssniff® 1554R/M-H maintenance diet; crude protein: 19.3%, crude fat: 3.3%, crude fiber: 4.4%, crude ash: 6.0%, starch: 36.1%, sugar: 3.1%) or chow containing 0.1% UDCA (w/w) (Sigma-Aldrich, St Louis, MO). Mice had unlimited access to food and drinking water during the experiment and were housed under climate-controlled conditions (21 °C) with a 12-h light/dark cycle. Food intake was monitored during pregnancy and suckling.

Dams were between 7 and 22 weeks of age during breeding and received their diet at least 4 days prior to mating. Either one or two females were co-housed with one male for 4 consecutive day/night cycles and housed individually after the 4th night. Pregnancy was confirmed between gestational days 9.5 and 13.5. Of all included female mice in this study, 88% were pregnant after 1 or 2 rounds of mating.

At the age of 3 weeks, WT and *Cyp2c70*^*-/-*^ offspring and their dams were sacrificed and blood and organs were collected as described below. *Cyp2c70*^*+/-*^ offspring were excluded from this study since their phenotype is indistinguishable from WT.^23^ In order to determine the long-term effects of perinatal UDCA exposure, part of the offspring was weaned to a non-UDCA-containing chow diet and housed individually at the age of 4 weeks. Blood samples from the tail vein were taken at 1 and 3 weeks after weaning, thus at the age of 5 and 7 weeks, respectively, to determine plasma UDCA concentrations. At the age of 8 weeks, these mice were sacrificed by cardiac puncture under isoflurane anesthesia. The liver was excised and the large lobe was immediately fixed in 4% formalin for histology. The remainder of the liver, the small intestine, and plasma were snap-frozen in liquid nitrogen and stored at −80 °C until further analyses.

All animal experiments were executed in accordance with the Dutch law and approved by the Dutch Central Committee for Animal Experiments, as well as by the Animal Welfare Body of the University of Groningen.

### Plasma parameters

Alanine aminotransferase (ALT), aspartate aminotransferase (AST), alkaline phosphatase (ALP), and bilirubin in plasma were quantified using a Cobas 6000 analyzer with standard reagents (Roche Diagnostics, Netherlands). BAs in plasma were measured with ultra (U)HPLC-MS/MS on a Nexera X2 UHPLC system (Shimadzu, Kyoto, Japan), coupled to a SCIEX QTRAP 4500 MD triple quadrupole mass spectrometer (SCIEX, Framingham, MA) and quantified using stable isotopically labeled internal standards.^23^

### Liver histology

Formalin-fixed paraffin-embedded liver sections (4 µm) were stained with hematoxylin and eosin to assess liver morphology. In order to visualize collagen deposition, Sirius Red/Fast Green staining was performed according to standard protocols. Cholangiocytes were stained using an anti-Cytokeratin 19 (CK19) rabbit monoclonal antibody (ab52625; Abcam, Cambridge, UK). Photomicrographic images were obtained using a Hamamatsu NanoZoomer (Hamamatsu Photonics, Almere, the Netherlands). Stained areas were quantified with Image J software^26^ and expressed as a percentage of the total image field.

### Hepatic gene expression

Snap-frozen livers were ground and total RNA was isolated by TRI-Reagent (Sigma-Aldrich, St. Louis, MO) according to the manufacturer’s protocol. RNA was reverse-transcribed using Moloney Murine Leukemia Virus Reverse Transcriptase (Life Technologies, Bleiswijk, The Netherlands) and Random Nonamers (Sigma-Aldrich). Real-time quantitative PCR was performed on a QuantStudio-3 system (Applied Biosystems, Foster City, CA) using TaqMan primer-probe combinations. Relative gene expression levels were calculated based on a dilution curve. All gene expression levels were normalized to 18S and further normalized to the average expression of WT pups from chow-fed dams, which served as the reference group for the 3-week-old mice. Female WT offspring from chow-fed dams served as the reference group in 8-week-old mice.

### Statistical analyses

Statistical analyses between two groups were performed by Mann–Whitney *U* tests (GraphPad Software, San Diego, CA, version 8). Multiple comparisons were analyzed using the Kruskal–Wallis *H* test, followed by Conover post hoc comparisons with Brightstat online software.^27^ Differences were considered statistically significant when *p* values were <0.05.

## Results

### UDCA treatment of dams does not affect litter size and body weight of their offspring

**Fig. 1:**
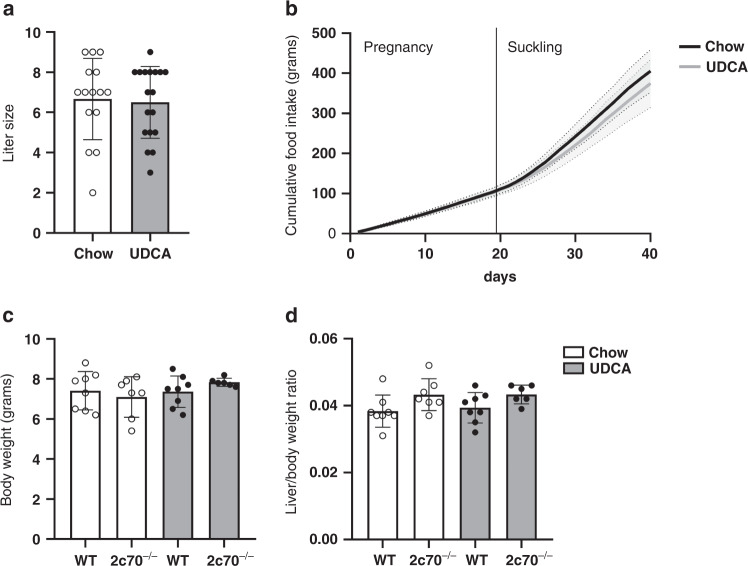
UDCA treatment of dams does not affect litter size and body weight of their offspring. **a** Litter sizes in the different treatment groups. **b** Cumulative food intake during pregnancy (19 days) and suckling (21 days) on either chow diet (*n* = 15) or UDCA diet (*n* = 18). **c** Body weights and **d** liver weights of 3-week-old WT and *Cyp2c70*^*-/-*^ pups of chow-fed and UDCA-fed dams. Dots (**a**, **c**, and **d**) represent individual values with mean and SD, whereas lines (**b**) represent mean values per diet group with SD.

In order to determine whether macronutrient intake during pregnancy and suckling differed between dams fed a regular chow diet or a chow diet supplemented with 0.1% UDCA, we monitored their food intake over time. Since dams with larger litters need more nutrients to provide sufficient energy for their pups, we evaluated the food intake of 15 dams on the chow diet and 18 dams on UDCA diet with a similar average litter size of 6.7 and 6.5 pups per litter, respectively, varying between 2 and 9 pups per litter in both diet groups (Fig. 1a).

Over the course of the experiment, the cumulative food intake during pregnancy and suckling was similar between chow- and UDCA-fed dams (Fig. 1b). In both groups, food intake during pregnancy remained relatively stable with an average of ~5.5 g/day (data not shown). After delivery, food intake of dams increased in both groups to peak at 2 weeks postpartum and gradually decreased thereafter. No significant differences were found in daily food intake between the diet groups. These results indicate that any differences found between pups from dams of either diet group are not caused by differences in macronutrient intake and are therefore most likely directly related to UDCA exposure.

WT and *Cyp2c70*^*-/-*^ pups from chow-fed as well as from UDCA-fed dams were sacrificed at the age of 3 weeks to assess the effects of perinatal UDCA treatment on features of neonatal cholestasis and liver pathophysiology. Genotypes of pups were according to normal Mendelian distribution in both diet groups (data not shown). The heterozygous offspring, accounting for ~50% of pups, was not included in the study because *Cyp2c70*^+/-^ mice have previously been shown to be phenotypically indistinguishable from WT.^23^ There were no differences between sexes within the 4 groups for any of the parameters that were determined at the age of 3 weeks. Therefore, in the results discussed below, data of males and females were combined for offspring from the same treatment groups and genotypes. Importantly, no differences were found in body weights (Fig. 1c) and liver weights (Fig. 1d) between WT and *Cyp2c70*^*-/-*^ pups or between pups from chow-fed dams and UDCA-fed dams at this age.

### Features of neonatal cholestasis in 3-week-old *Cyp2c70*^*-/-*^ pups are prevented by the addition of UDCA to the diet of their dams

**Fig. 2:**
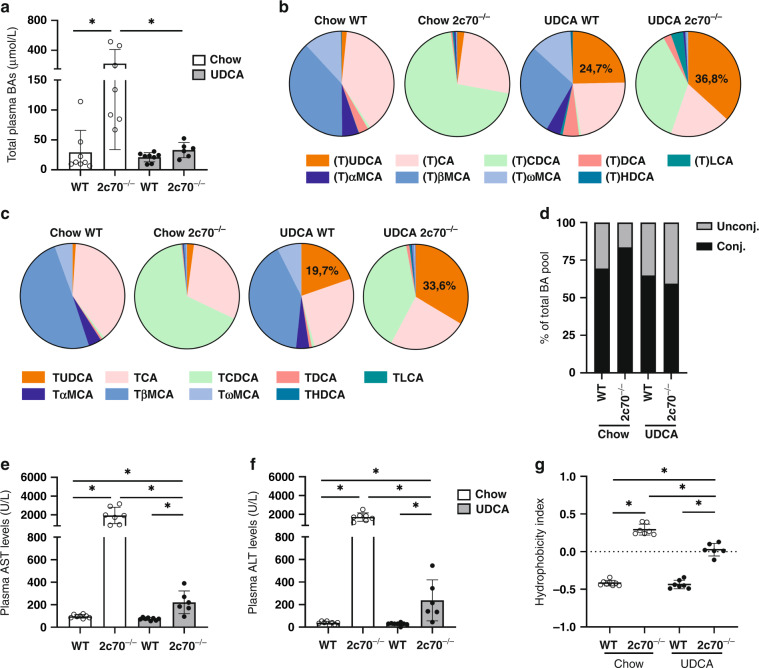
Perinatal UDCA exposure prevents the development of features of neonatal cholestasis in 3-week-old *Cyp2c70*^*-/-*^ pups. **a** Total plasma BA levels in 3-week-old pups. **b** Composition of total BAs in plasma and **c** bile. **d** Percentage of conjugated and unconjugated BAs in plasma. **e** Plasma AST and **f** ALT levels. **g** Hydrophobicity index of biliary BAs. Dots represent individual values with mean and SD. *P < 0.05 using Kruskal–Wallis *H* test followed by Conover post hoc comparisons. ALT alanine aminotransferase, AST aspartate aminotransferase, BA bile acids, conj conjugated BAs, unconj unconjugated BAs.

In line with previous observations,^23^ newborn *Cyp2c70*^*-/-*^ mice displayed features of neonatal cholestasis. *Cyp2c70*^*-/-*^ mice of 3 weeks old showed significantly higher total BA levels in plasma than WT mice of the same age (Fig. 2a). However, when their dams were fed the UDCA-containing diet, plasma BAs in their *Cyp2c70*^-/-^ pups were reduced to normal levels, i.e., concentrations were comparable to those found in WT pups. The percentages of UDCA as part of total BAs in plasma and bile were increased in pups from UDCA-fed dams as compared to pups from chow-fed dams, with averages of 24.7% (WT) and 36.8% (*Cyp2c70*^*-/-*^) in plasma (Fig. 2b) and 19.7% (WT) and 33.6% (*Cyp2c70*^*-/-*^) in bile (Fig. 2c), while UDCA constituted <5% of BAs in plasma and bile of pups from chow-fed dams. No significant differences were observed in the ratio of conjugated versus unconjugated BAs in plasma (Fig. 2d). In addition to high BA levels, 3-week-old *Cyp2c70*^*-/-*^ mice showed markedly increased transaminase levels, indicating liver damage. Plasma AST and ALT (Fig. 2e, f), as well as ALP (Supplementary Fig. S1a), were significantly increased in *Cyp2c70*^*-/-*^ pups compared to WT. Interestingly, plasma transaminase levels in *Cyp2c70*^*-/-*^ pups from UDCA-fed dams were strongly decreased compared to *Cyp2c70*^*-/-*^ pups from chow-fed dams though still somewhat higher than in WT pups, whereas ALP levels were normalized. Plasma bilirubin levels displayed a similar pattern although with substantial variation in *Cyp2c70*^*-/-*^ pups from chow-fed dams (Supplementary Fig. S1b). The improved liver function parameters in *Cyp2c70*^*-/-*^ pups from UDCA-fed dams coincided with reduced hydrophobicity of their biliary BAs as compared to *Cyp2c70*^*-/-*^ pups from chow-fed dams. However, the hydrophobicity index was still higher in *Cyp2c70*^*-/-*^ pups from UDCA-fed dams than in WT pups from dams that were either fed chow or the UDCA-supplemented diet (Fig. 2g).

### Perinatal UDCA exposure corrects dysregulated expression of genes encoding BA transporters and synthesis enzymes and reduces hepatic expression of pro-inflammatory cytokines

**Fig. 3:**
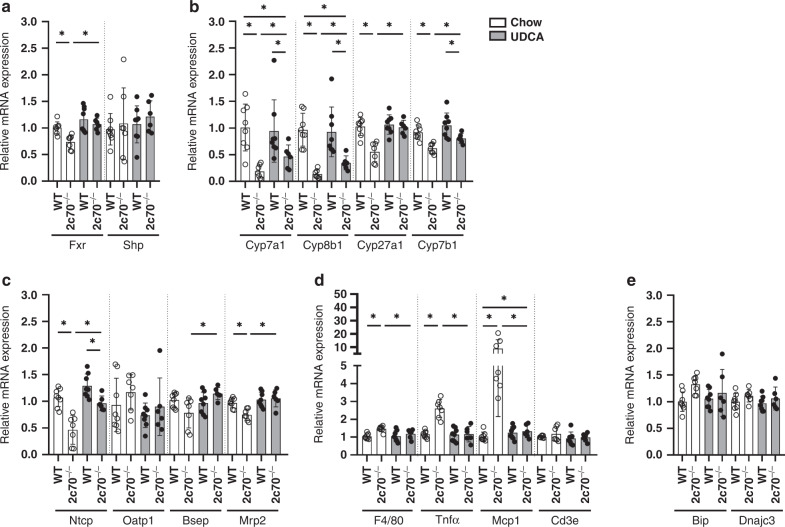
Hepatic expression of BA-related genes and pro-inflammatory cytokines is restored in *Cyp2c70*^*-/-*^ pups from UDCA-fed dams. Hepatic expression of genes involved in **a** BA signaling, **b** BA synthesis, **c** BA transport, **d** inflammation, and **e** endoplasmic reticulum stress in 3-week-old pups. Data are depicted as individual values with mean and SD. *P < 0.05 using Kruskal–Wallis *H* test followed by Conover post hoc comparisons.

In order to evaluate whether perinatal UDCA exposure affects BA homeostasis or inflammation, we assessed the expression patterns of BA-related genes and pro-inflammatory cytokines in the pups. *Cyp2c70*^*-/-*^ pups from chow-fed dams possess a more hydrophobic BA pool containing potent FXR agonists, including CDCA, which may lead to altered expression of FXR-controlled genes. However, the expression of the FXR target small heterodimer partner (*Shp/Nr0b2*) remained unchanged in pups from all groups (Fig. 3a), while the expression of *Fxr* (*Nr1h4*) itself was decreased in *Cyp2c70*^*-/-*^ pups from chow-fed dams compared to their WT littermates and was restored to normal levels upon addition of UDCA to the diet of their dams.

Next, gene expression of BA synthesis enzymes catalyzing key steps in the classic (*Cyp7a1, Cyp8b1*) and the alternative (*Cyp27a1, Cyp7b1*) pathway was measured (Fig. 3b). Despite a decrease in *Fxr* and similar *Shp* expression, *Cyp2c70*^*-/-*^ pups from chow-fed dams showed decreased expression of all measured BA synthesis genes. Expression levels of *Cyp7a1, Cyp8b1*, and *Cyp7b1* were partially restored in *Cyp2c70*^*-/-*^ pups from UDCA-fed dams, while the expression of *Cyp27a1* was completely normalized.

Furthermore, hepatic mRNA expression of transporters that mediate uptake of BAs from the portal vein into the hepatocytes (*Ntcp/Slc10a1* and *Oatp1/Slco1a1*), as well as expression of transporters that export BAs from hepatocytes into the bile (*Bsep/Abcb11* and *Mrp2/Abcc2*) was measured (Fig. 3c). No differences in *Oatp1* expression were present. However, compared to their WT littermates, the expression of *Ntcp* was significantly decreased in *Cyp2c70*^*-/-*^ pups from chow-fed dams, while its expression was restored in *Cyp2c70*^*-/-*^ pups from UDCA-fed dams. Similarly, compared to WT, *Mrp2* and *Bsep* transcripts were decreased and tended to decrease, respectively, in *Cyp2c70*^*-/-*^ pups from chow-fed dams and were restored in *Cyp2c70*^*-/-*^ pups from UDCA-fed dams. These findings suggest an overall improvement of the capacity to transport BAs from the portal vein into the bile in *Cyp2c70*^*-/-*^ pups from UDCA-fed dams as compared to *Cyp2c70*^-/-^ pups from dams that did not receive treatment.

In addition, we investigated the potential involvement of inflammatory pathways and ER stress in UDCA-associated improvement of liver function. Expression of macrophage marker *F4/80 (Adgre1)* and the inflammation markers *Tnfα* and monocyte attractant protein *Mcp1 (Ccl2)* was significantly increased in *Cyp2c70*^*-/-*^ pups from chow-fed dams and was normalized in *Cyp2c70*^*-/-*^ pups from UDCA-fed dams (Fig. 3d), whereas expression of T-cell marker *Cd3e* remained unaffected in all groups. Moreover, no significant differences in the expression of ER stress markers *Bip (Hspa5)* and *Dnajc3 (Hsp40)* were observed between the groups (Fig. 3e).

### Perinatal UDCA exposure prevents cholangiopathy in *Cyp2c70*^*-/-*^ pups

**Fig. 4:**
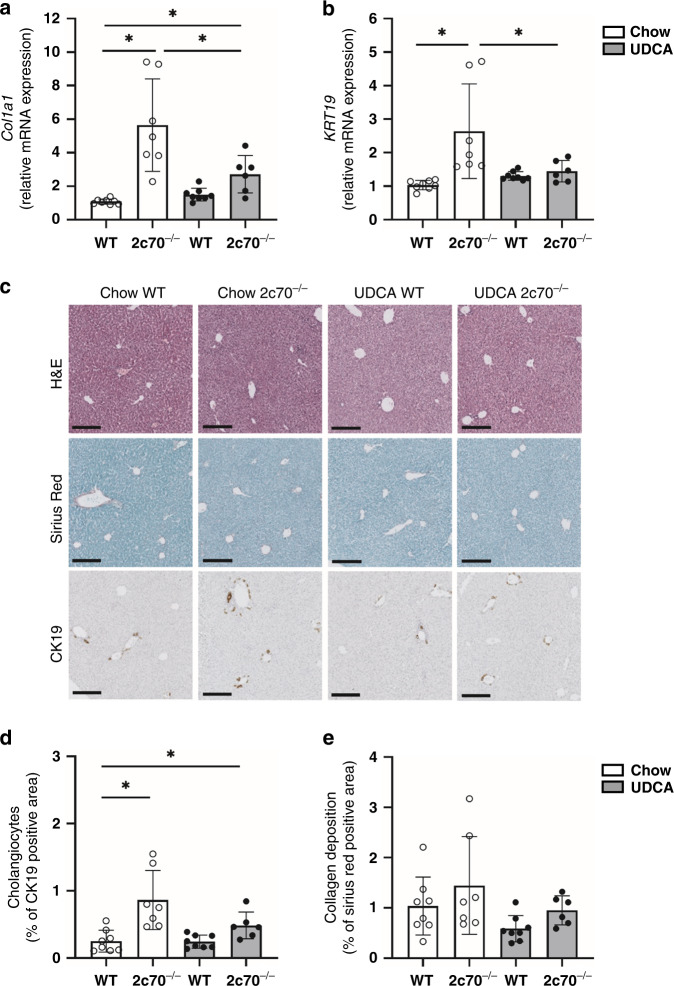
Perinatal UDCA treatment prevents fibrogenesis and cholangiopathy in *Cyp2c70*^*-/-*^ pups. Hepatic mRNA expression levels of **a** *Col1a1* and **b** *Krt19* in 3-week-old pups. **c** Representative histological photomicrographs of livers stained with hematoxylin and eosin (H&E), Sirius Red/Fast Green, and anti-CK19. Quantification of **d** CK19 positively stained areas and **e** collagen deposition in livers of the 3-week-old pups. Bars represent 200 µm. *P < 0.05 using Kruskal–Wallis *H* test followed by Conover post hoc comparisons.

Next, we evaluated markers of fibrogenesis and ductular reactions. Hepatic expression of collagen type I alpha-1 *(Col1a1)* was substantially increased in *Cyp2c70*^*-/-*^ pups from chow-fed dams as compared to WT pups (Fig. 4a), indicating augmented fibrogenesis in these pups. Hepatic *Col1a1* expression levels in *Cyp2c70*^*-/-*^ pups from UDCA-fed dams were restored, but remained significantly elevated compared to WT pups. Moreover, hepatic mRNA expression of the cholangiocyte marker keratin-19 (*Krt19*, encoding the cholangiocyte marker CK19) was significantly increased in *Cyp2c70*^*-/-*^ pups from chow-fed dams and completely normalized in pups from UDCA-fed dams (Fig. 4b). Histological quantification confirmed an increase in CK19-positive cells in *Cyp2c70*^*-/-*^ pups from chow-fed dams compared to WT, which tended to be reduced in *Cyp2c70*^-/-^ pups from dams fed the UDCA-containing diet (Fig. 4c, d). Only limited collagen deposition was observed in these 3-week-old pups, without significant differences between the groups (Fig. 4c, e). No differences in overall liver morphology were evident between *Cyp2c70*^*-/-*^ pups from chow- and UDCA-fed dams (Fig. 4c).

In summary, the combined data obtained from 3-week-old pups demonstrate that perinatal exposure to UDCA prevents the development of liver injury in *Cyp2c70*^*-/-*^ mice at this age.

### Perinatal UDCA exposure does not confer long-lasting protection from liver pathology in *Cyp2c70*^*-/-*^ mice

**Fig. 5:**
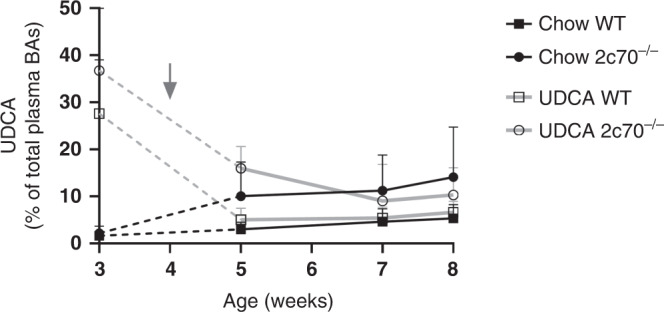
UDCA is cleared rapidly from the circulating BA pool. UDCA as a percent of total plasma BAs (*n* = 6–18 samples per group per time point). The arrow indicates the age at which the pups were weaned and transferred to a non-UDCA-containing chow diet. BAs bile acids.

To investigate potential long-lasting beneficial effects of UDCA exposure early in life, WT and *Cyp2c70*^*-/-*^ pups from chow- and UDCA-fed dams were weaned to a non-UDCA-containing diet and housed individually at the age of 4 weeks. In order to determine the time window in which the additional UDCA is cleared from the circulating BA pool in pups from UDCA-fed dams, UDCA levels in plasma were determined at multiple timepoints after weaning. Already one week after weaning, the amounts of UDCA in plasma of *Cyp2c70*^*-/-*^ pups from UDCA-fed dams had dropped to levels that were fairly comparable to those found in pups from chow-fed dams, indicating that UDCA is cleared from the enterohepatic circulation within days after discontinuation of treatment. After that, the levels of UDCA remained relatively stable (Fig. 5).

**Fig. 6:**
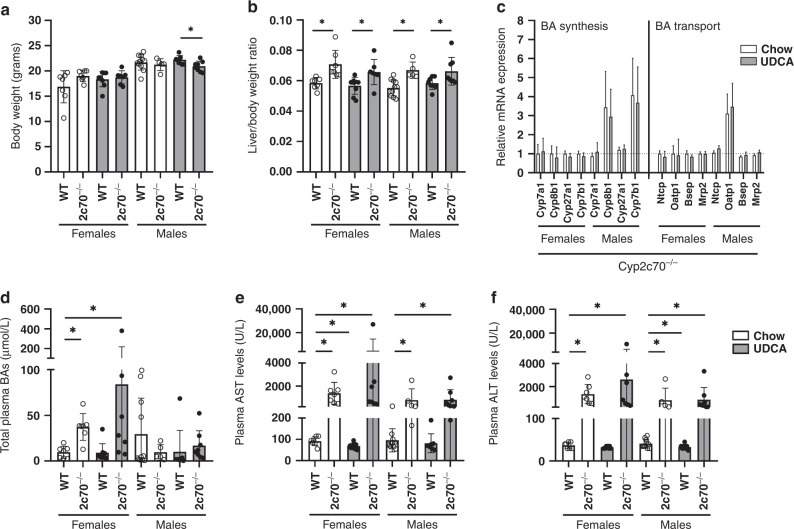
Perinatal UDCA exposure does not protect against cholangiopathy development in adulthood. **a** Body weights and **b** liver weights of 8-week-old WT and *Cyp2c70*^*-/-*^ offspring from chow- and UDCA-fed dams. **c** Hepatic gene expression levels of *Cyp2c70*^*-/-*^ offspring from chow- and UDCA-fed dams. Only *Cyp2c70*^*-/-*^ mice are shown, and expression of female WT offspring from chow-fed dams was used as a reference group, see the dotted line. **d** Total BA concentrations, **e** AST, and **f** ALT levels in the plasma of *Cyp2c70*^*-/-*^ offspring from chow- and UDCA-fed dams. For panels **a**, **b**, **d**, **e**, and **f**, dots represent individual values with mean and SD, while only mean and SD are depicted in panel **c**. *P < 0.05 using Kruskal–Wallis *H* test followed by Conover post hoc comparisons. ALT alanine aminotransferase, AST aspartate aminotransferase, BA bile acids.

Based on the relatively rapid clearance of UDCA from the BA pool of the pups after weaning, it was decided to study the long-lasting effects of perinatal UDCA treatment at the age of 8 weeks. For all parameters described below, males and females were analyzed separately, since sex has been shown to impact liver pathophysiology in *Cyp2c70*^*-/-*^ mice.^23^ No major differences in body weight development from 4 until 8 weeks of age were observed between the groups (Fig. 6a and Supplementary Fig. S2). Liver weights were significantly increased in male and female *Cyp2c70*^*-/-*^ mice compared to WT. However, no effect of perinatal UDCA treatment was observed at this age (Fig. 6b). Hepatic expression of genes encoding BA transporters and BA synthesis enzymes in *Cyp2c70*^*-/-*^ offspring of chow-fed dams and UDCA-fed dams was similar (Fig. 6c). Total plasma BA concentrations were significantly increased in female but not in male *Cyp2c70*^*-/-*^ mice compared to WT littermates, yet were comparable in *Cyp2c70*^*-/-*^ offspring that had been perinatally exposed to UDCA and in offspring that had not been exposed to UDCA (Fig. 6d). Transaminase levels in plasma were significantly increased in both male and female *Cyp2c70*^*-/-*^ compared to WT offspring (Fig. 6e, f), but no effects of perinatal UDCA exposure were detected. Finally, no changes in hepatic collagen deposition, CK19-positive cells, or liver morphology were observed between offspring of chow-fed or UDCA-fed dams (data not shown).

Taken together, although perinatal exposure to UDCA conferred protection from hypercholemia and cholangiopathy in 3-week-old *Cyp2c70*^*-/-*^ pups, livers of *Cyp2c70*^*-/-*^ offspring of UDCA-fed dams were phenotypically indistinguishable from offspring of untreated dams by the age of 8 weeks. These findings indicate that perinatal UDCA exposure does not translate into long-lasting effects on liver pathophysiology in these mice after discontinuation of the treatment.

## Discussion

*Cyp2c70*^*-/-*^ mice that are devoid of mouse/rat-specific MCAs show features of neonatal cholestasis, including elevated plasma BAs and transaminases.^23^ In the current study, we show that the addition of UDCA to the diet of dams during gestation and lactation prevents elevation of plasma BAs and transaminases in their 3-week-old *Cyp2c70*^*-/-*^ offspring. However, perinatal exposure to UDCA did not confer long-lasting protection against the development of liver pathology in *Cyp2c70*^*-/-*^ offspring after discontinuation of treatment.

Even though BAs are not required for lipid uptake in the human fetus, development of the BA synthesis and transport systems starts during fetal development.^28,29^ After delivery, the activities of these systems rapidly increase in both humans and murine newborns.^30,31^ Similar to adults, the primary BAs CA and CDCA are synthesized in fetal hepatocytes. However, differences in BA synthesis between fetuses, newborns, and adults do exist. The classical BA synthesis pathway produces most primary BAs in human adults, while in fetuses a significant contribution of the “Yamasaki pathway” has been postulated.^29^ BA synthesis via this pathway mainly produces CDCA, with 3β,7α-dihydroxy-5-cholenoic acid as an intermediate.^32^ In addition, unusual BA species, such as 1β- and 6α-hydroxylated BAs, have been found in the BA pool of human fetuses and newborns.^28,33^ It is currently unknown whether these BAs fulfill specific functions. Recently, it was described that BAs drive the gut microbiota maturation,^34^ underlining the important role of BAs during early development. Lastly, while BAs are mainly conjugated to glycine in human adults, taurine conjugation predominates in fetuses and newborns.^28,35,36^

In humans, fetal BAs are exported through the placenta in order to be cleared from the circulation by the maternal liver.^37–39^ Vice versa, it has been reported that BAs from the maternal serum cross the placenta and end up in the circulation of the fetus.^37^ Transporters from the MRP-, ABC- and OATP families have been identified in the placenta and have been proposed to be involved in BA transport between mother and fetus.^37,40^ We did not quantify UDCA levels in the fetuses in the current study, but in vitro studies have shown that UDCA can be transported across the placenta bidirectionally.^41^ It is therefore conceivable that UDCA treatment of the dams during gestation leads to elevated concentrations of UDCA in the circulation and livers of *Cyp2c70*^*-/-*^ fetuses.

It has been described that fetal exposure to high BA levels leads to a higher risk of developing metabolic disease in later life, including obesity, increased hepatic inflammatory responses, and insulin resistance.^42^ Studies showed that UDCA treatment during pregnancy can prevent this predisposition effect in both human and animal models.^43–45^ Intriguingly, in a study by Pataia et al., even offspring of male mice with cholestasis were shown to be more susceptible to adiposity-related metabolic diseases in later life, which could be prevented by UDCA treatment of the father.^46^ Several studies in humans have evaluated the efficacy of UDCA treatment during pregnancy and report that UDCA is safe for the offspring without any adverse effects in later life.^47,48^ Data on the effects of UDCA treatment in newborns with neonatal cholestasis have been less conclusive.^49,50^ In the current study, we show that perinatal exposure to UDCA had beneficial effects on features of cholestasis and liver pathology in 3-week-old *Cyp2c70*^-/-^ mice. Prophylactic UDCA administration may be beneficial for neonates that are at risk of developing cholestasis, such as preterm neonates that receive parenteral nutrition (PN). Long-term PN is applied in extreme preterm neonates and in neonates that cannot receive enteral nutrition due to illness or surgery. PN is associated with an increased risk of developing cholestasis, i.e., parenteral nutrition-associated cholestasis (PNAC), which in turn is associated with an increased risk of complications and mortality.^51^ However, the effects of UDCA administration on PNAC in preterm infants have been studied mainly in small cohorts and are therefore incompletely established.^52–54^ Furthermore, as discussed above, exposure to high BA levels early in life is associated with an increased risk of developing metabolic diseases in later life.^42^ Prevention of PNAC by UDCA in preterm neonates might therefore reduce the risk of metabolic diseases in later life. However, the metabolic consequences of perinatal UDCA treatment were not investigated in the current study.

In our study, we did not find any indications of long-lasting effects of perinatal exposure to UDCA. However, it may be that UDCA has long-term effects on parameters that were not measured in this study, e.g., metabolic parameters.

In this study, dams received UDCA not only during gestation but also during the lactation period. Milk production in dams peaks when their pups are around 2 weeks of age and it has been shown that BAs are transported from the blood into milk via BA transporters in the mammary glands.^55,56^ BAs can be detected in human milk, but there is no evidence of naturally occurring UDCA in milk.^57^ Yet, UDCA could be detected in the milk of mothers treated with UDCA during their pregnancy,^58^ indicating that UDCA can indeed be transported from the blood into milk. These findings support the notion that UDCA-fed dams in the current study transfer UDCA to their pups via milk during the suckling period. Since pups gradually start consuming solid food from approximately 2 weeks of age onwards, pups from UDCA-fed dams may have ingested some additional UDCA directly via the diet.

Despite high plasma concentrations of the strong FXR agonist CDCA in *Cyp2c70*^*-/-*^ pups, the current study shows no effect of *Cyp2c70*-deficiency on the expression of its direct target genes *Shp* and *Bsep* in the liver. Nevertheless, hepatic mRNA levels of BA synthesis enzymes, including those of the rate-controlling enzyme *Cyp7a1*, are decreased in *Cyp2c70*^*-/-*^ pups, suggesting inhibition of BA synthesis via mechanisms other than FXR signaling. Instead, inhibition of the expression of BA synthesis enzymes as well as the BA importer *Ntcp* in 3-week-old *Cyp2c70*^-/-^ pups, might be driven by inflammatory pathways. Pro-inflammatory cytokines have been shown to decrease *CYP7A1* expression in human primary hepatocytes^59^ and to decrease *Ntcp* expression in primary rat hepatocytes.^60^ In our study, we observed a substantially decreased expression of pro-inflammatory cytokines in livers of *Cyp2c70*^*-/-*^ pups from UDCA-fed dams compared to those originating from dams fed regular chow, which coincided with normalization of the expression of BA synthesis enzymes and *Ntcp*. Taken together, these findings suggest a direct role for inflammatory signaling pathways in the development of neonatal cholestasis in *Cyp2c70*^*-/-*^ pups, which can be modulated by UDCA. The absence of clear FXR activation in *Cyp2c70*^*-/-*^ mice, despite the high abundance of CDCA in the BA pool of these mice, can possibly be explained by the relatively low sensitivity of murine FXR for CDCA as compared to human FXR,^61^ underscoring the presence of substantial differences between FXR signaling in humans and mice.

In conclusion, in the current study we show that the addition of UDCA to the diet of the dams during gestation and suckling leads to the incorporation of UDCA into the circulating BA pool of *Cyp2c70*^-/-^ pups, leading to a considerably more hydrophilic composition. The increased hydrophilicity conceivably effectively counteracts the impact of high concentrations of hydrophobic BAs like CDCA on liver function in *Cyp2c70*^*-/-*^ pups, while anti-inflammatory effects of UDCA likely also contribute to the improvements of liver physiology upon perinatal exposure in these mice. Despite the robust direct effects on neonatal cholestasis and liver pathology in the suckling pups, the effects of perinatal UDCA exposure are not maintained after discontinuation of treatment.

## Supplementary information

Supplementary material

## Data Availability

All data generated or analyzed during this study are included in this published article.
