# Supplementary material for: Perinatal exposure to UDCA prevents neonatal cholestasis in Cyp2c70-/- mice with human-like bile acids
Source: Pediatr Res. 2022 Sep 23;93(6):1582–90. doi: 10.1038/s41390-022-02303-5 (PMC10172110; doi:10.1038/s41390-022-02303-5)
Supplement: Supplementary file 1 — Supplementary material [file 41390_2022_2303_MOESM1_ESM.pdf]

Supplementary material

Perinatal exposure to UDCA prevents neonatal cholestasis in *Cyp2c70*$^{-/-}$ mice with human-like bile acids

Hilde D. de Vries[1,2], Anna Palmiotti[3], Rumei Li[3], Milaine V. Hovingh[3], Niels L. Mulder[3], Martijn Koehorst[1], Vincent. W. Bloks[3], Tim van Zutphen[2,3], Folkert Kuipers[1,3], Jan Freark de Boer[1,3,*]

*Correspondence to Jan Freark de Boer, PhD, University Medical Center Groningen, Department of Pediatrics, CA84, Hanzeplein 1, 9713 GZ, Groningen, the Netherlands. Phone: +31 50 361 4865, fax: +31(0)503611746, email address: j.f.de.boer@umcg.nl

Affiliations

1. Department of Laboratory Medicine, University of Groningen, University Medical Center Groningen, Groningen, The Netherlands
2. Faculty Campus Fryslân, University of Groningen, Leeuwarden, The Netherlands
3. Department of Pediatrics, University of Groningen, University Medical Center Groningen, Groningen, The Netherlands

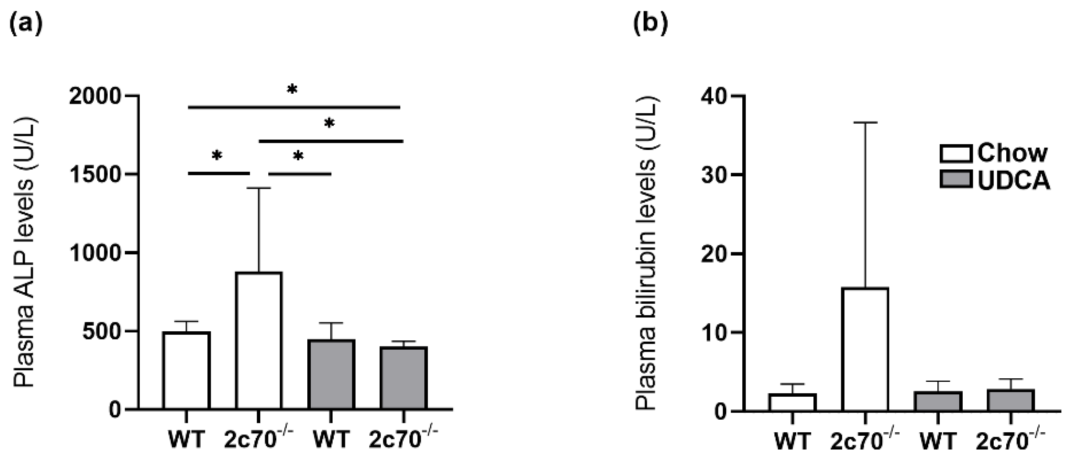

Supplementary Fig. S1: Plasma alkaline phosphatase (a) and bilirubin (b) levels in 3-week old *Cyp2c70$^{-/-}$* pups and wild type controls. * P <0.05 using Kruskal Wallis H test followed by Conover *post hoc* comparisons (n=6-8 mice/group). ALP, alkaline phosphatase.

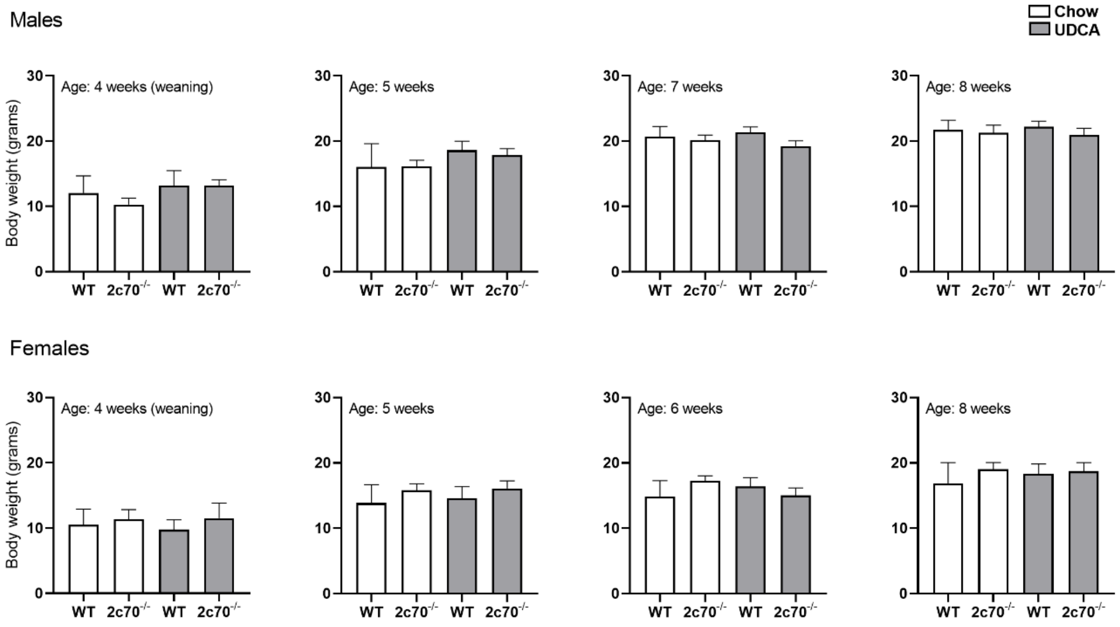

Supplementary Fig. S2: Body weights of *Cyp2c70*$^{-/-}$ mice and wild type controls after weaning. Male and female *Cyp2c70*$^{-/-}$ and wild type pups from UCDA- and chow-fed dams were weaned to a non-UDCA-containing chow diet at the age of 4 weeks and body weights were monitored at the ages indicated in the figures**.** No significant differences between any of the groups were detected at any of the indicated ages using the Kruskal Wallis H test (n=3-11 mice, depending on group and time point).
